# Supplementary material for: Stimuli-Responsive Thiomorpholine Oxide-Derived Polymers with Tailored Hydrophilicity and Hemocompatible Properties
Source: Molecules. 2022 Jun 30;27(13):4233. doi: 10.3390/molecules27134233 (PMC9268026; doi:10.3390/molecules27134233)
Supplement: Supplementary file 1 [file molecules-27-04233-s001.zip › molecules-1784821-supplementary.pdf]

Stimuli-Responsive Thiomorpholine Oxide-Derived Polymers with Tailored Hydrophilicity and Hemocompatible Properties

Supplementary Information

Laura Vasilica Arsenie,[1] Franziska Hausig,[2] Carolin Kellner,[2] Johannes C. Brendel,[2,3] Patrick Lacroix-Desmazes,[1] Vincent Ladmiral,[1,*] Sylvain Catrouillet[1,*]

[1]ICGM, University of Montpellier, CNRS, ENSCM, Montpellier, France

[2]Laboratory of Organic and Macromolecular Chemistry (IOMC), Friedrich Schiller University Jena, Jena, Germany

[3]Jena Center for Soft Matter (JCSM), Friedrich Schiller University Jena, Philosophenweg 7, 07743 Jena, Germany

Fig. S1. ^{1}H-NMR spectra in $CDCl_3$ of THMA (red traces) and THOXMA (blue traces) 2

Fig. S2. FTIR characterization of THMA (red traces) and THOXMA (blue traces) 3

Table S1: Experimental conditions for the synthesis of $P(THOXMA)_{100}$ homopolymer and statistical P(THOXMA$_n$-*stat*-HEMA$_m$) copolymers by RAFT polymerization 4

Fig. S3. Evolution of conversion of THOXMA over time for $P(THOXMA)_{100}$ (A), 1st order monomer kinetic plot (B) of RAFT homopolymerization of THOXMA 5

Fig. S4. Conversion of THOXMA (A) and HEMA (B) co-monomers used to prepare P(THOXMA$_n$-stat-HEMA$_m$) copolymers; First order monomers kinetic plot for RAFT statistical copolymerizations of THOXMA and HEMA (C, D) 6

Fig. S5. ^{1}H-NMR spectrum in DMSO-d6 of P(THOXMA$_{70}$-stat-HEMA$_{30}$) copolymer prepared by RAFT polymerization 7

Fig. S6. Titration curve of PTHOXMA (blue) and THOXMA (orange). 8

Fig. S7. Size evolution vs. temperature at pH=4, determined by dynamic light scattering. 9

Table S2: LCST values of THOXMA containing copolymers evaluated at pH=7.4 and pH=10 10

Table S3. pK_a values of monomer and corresponding polymer in water and 0.9% (wt%) NaCl solution 10

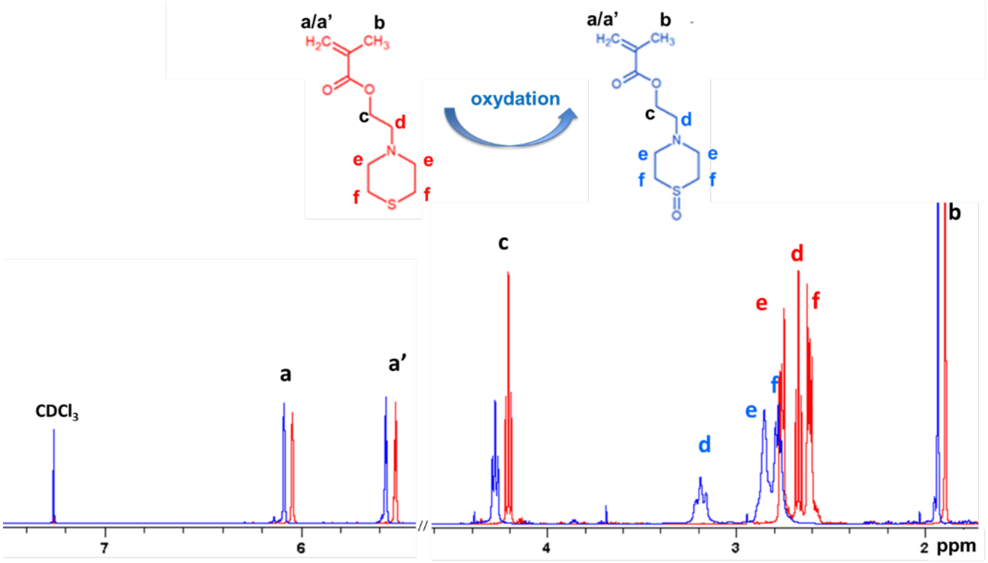

Figure S1. ^{1}H-NMR spectra in $CDCl_3$ of THMA (red traces) and THOXMA (blue traces).

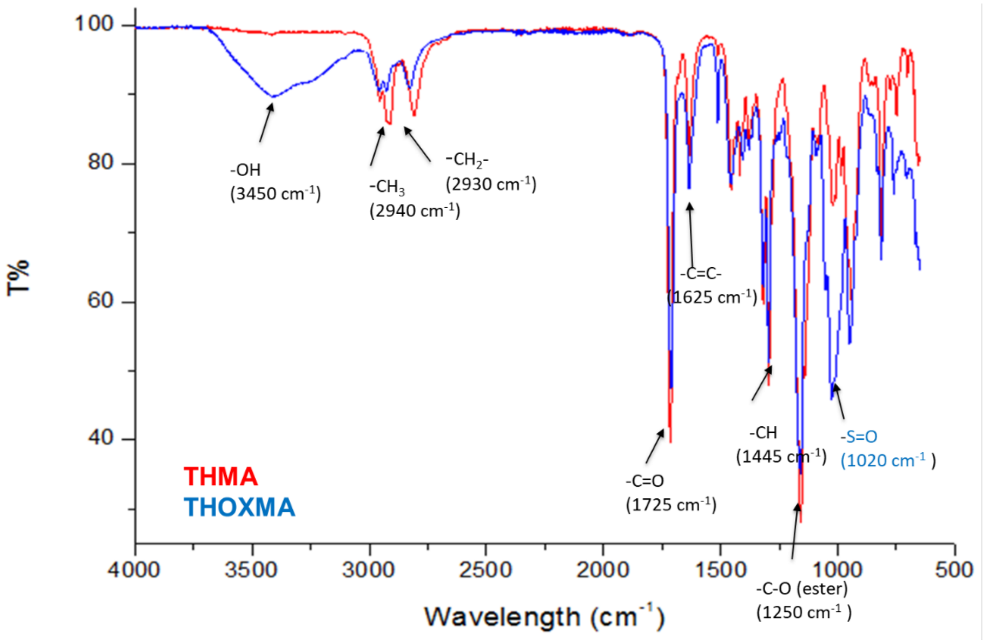

Figure S2. FTIR characterization of THMA (red traces) and THOXMA (blue traces).

Table S1. Experimental conditions for the synthesis of P(THOXMA)$_{100}$ homopolymer and statistical P(THOXMA$_n$-*stat*-HEMA$_m$) copolymers by RAFT polymerization.

Entry	Theoretical degree of polymerization (DP)	Theoretical molar fraction of monomers (%)		Experimental degree of polymerization (DP), determined by ^{1}H-NMR	Experimental molar fraction of monomers (%), determined by ^{1}H-NMR		Reaction yield (%)	[Monomer(s)]/ [CPDB]/[AIBN]	Reaction time (h)
		THOXMA	HEMA		THOXMA	HEMA			
P(THOXMA$_{100}$)	100	100	-	111	100	-	80	100/1/0.25	6
P(THOXMA$_{80}$-*stat*-HEMA$_{20}$)	100	80	20	80	78	22	80	100/1/0.25	4
P(THOXMA$_{50}$-*stat*-HEMA$_{50}$)	100	50	50	82	52	48	78	100/1/0.25	4
P(THOXMA$_{40}$-*stat*-HEMA$_{60}$)	100	40	60	81	43	57	82	100/1/0.25	4
P(THOXMA$_{35}$-*stat*-HEMA$_{65}$)	100	35	65	80	33	66	75	100/1/0.25	4
P(THOXMA$_{30}$-*stat*-HEMA$_{70}$)	100	30	70	82	27	73	83	100/1/0.25	4

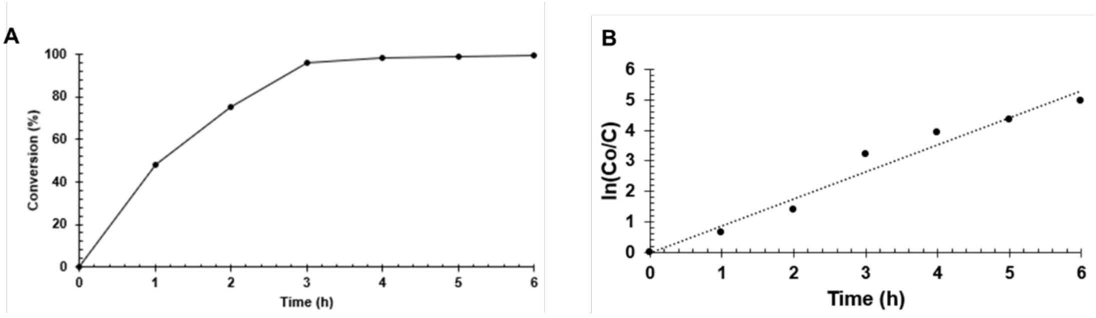

Figure S3. Evolution of conversion of THOXMA over time for P(THOXMA)$_{100}$ (A), 1st order monomer kinetic plot (B) of RAFT homopolymerization of THOXMA.

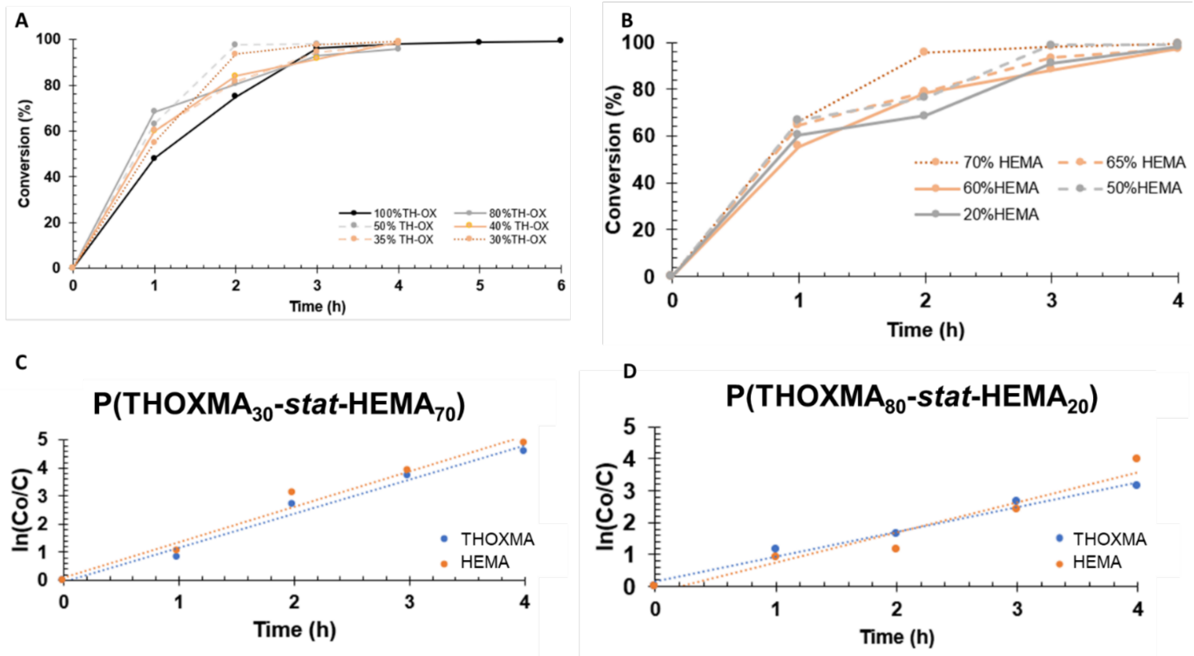

Figure S4. Conversion of THOXMA (A) and HEMA (B) co-monomers used to prepare P(THOXMA$_n$-stat-HEMA$_m$) copolymers; First order monomers kinetic plot for RAFT statistical copolymerizations of THOXMA and HEMA (C, D).

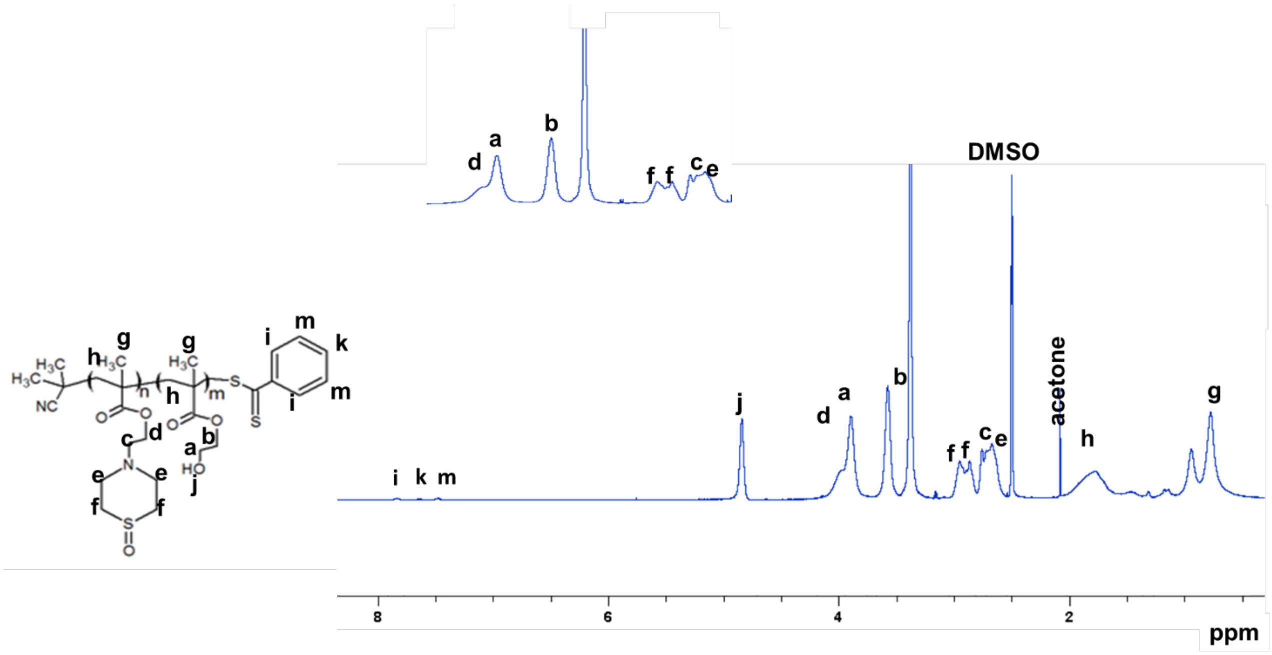

Figure S5. ^{1}H-NMR spectrum in DMSO-d6 of P(THOXMA$_{70}$-stat-HEMA$_{30}$) copolymer prepared by RAFT polymerization.

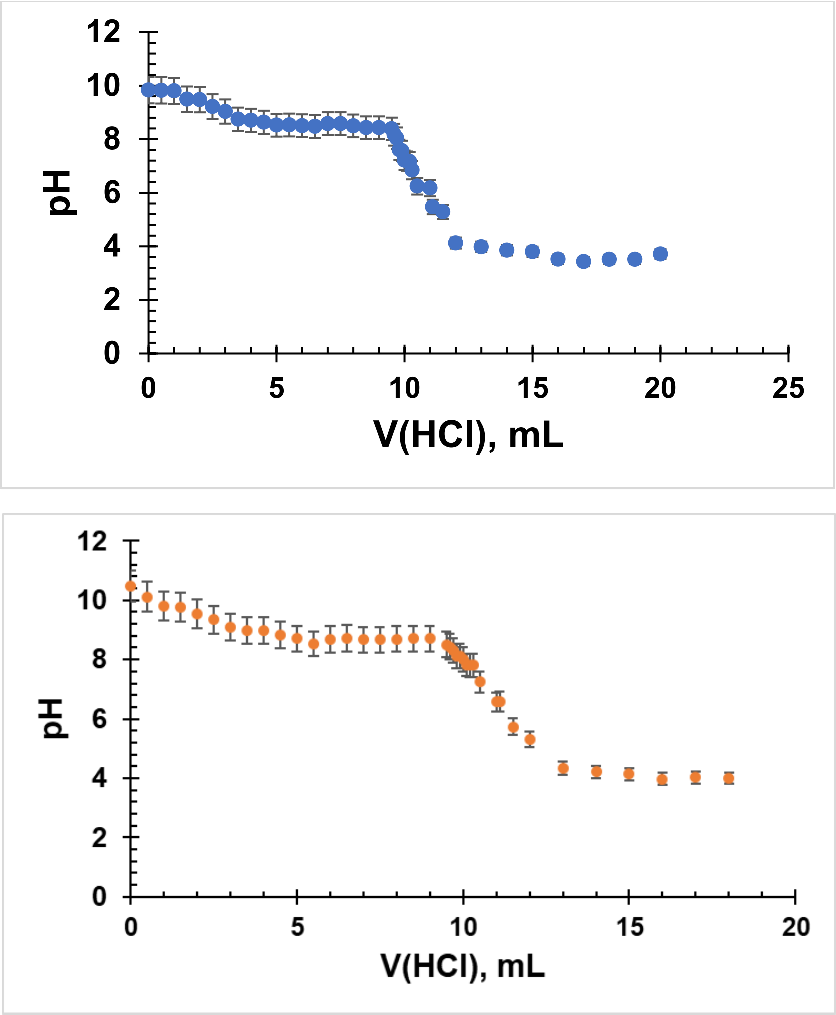

Figure S6. Titration curve of PTHOXMA (blue) and THOXMA (orange).

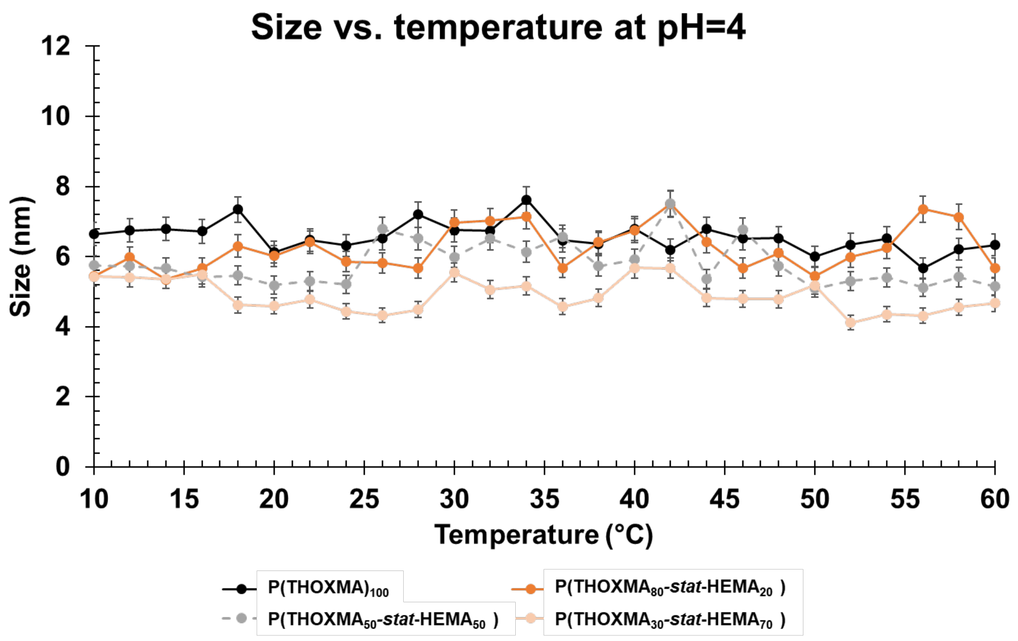

Figure S7. Size evolution vs. temperature at pH=4, determined by dynamic light scattering.

Table S2. LCST values of THOXMA containing copolymers evaluated at pH=7.4 and pH=10.

Entry	LCST(°C, at pH=7.4)	LCST(°C, at pH=10)
P(THOXMA$_{30}$-*stat*-HEMA$_{70}$)	42.0	36.0
P(THOXMA$_{35}$-*stat*-HEMA$_{65}$)	47.1	37.0
P(THOXMA$_{40}$-*stat*-HEMA$_{60}$)	49.5	37.2
P(THOXMA$_{50}$-*stat*-HEMA$_{50}$)	52.0	39.5
P(THOXMA$_{75}$-*stat*-HEMA$_{35}$)	56.0	52.0

Table S3. pK_a values of monomer and corresponding polymer in water and 0.9% (wt%) NaCl solution.

Entry	pK_a
THOXMA	5.42
PTHOXMA	5.57
PTHOXMA in NaCl	5.65
